# Supplementary material for: High-harmonic generation in Weyl semimetal β-WP2 crystals
Source: Nat Commun. 2021 Nov 8;12:6437. doi: 10.1038/s41467-021-26766-y (PMC8575912; doi:10.1038/s41467-021-26766-y)
Supplement: Supplementary file 1 — Supplementary information [file 41467_2021_26766_MOESM1_ESM.pdf]

Supplementary information of "High-harmonic generation in Weyl semimetal β-WP_2 crystals"

Yang-Yang Lv[1,&], Jinlong Xu[1,&,*], Shuang Han[1], Chi Zhang[1], Yadong Han[2], Jian Zhou[1], Shu-Hua Yao[1,*], Xiao-Ping Liu[3], Ming-Hui Lu[1], Hongming Weng[4], Zhenda Xie[1,*], Y. B. Chen[1,*], Jianbo Hu[2], Yan-Feng Chen[1] & Shining Zhu[1]

[1] National Laboratory of Solid State Microstructures, School of Physics, School of Electronic Science and Engineering, College of Engineering and Applied Sciences, and Collaborative Innovation Center of Advanced Microstructures, Nanjing University, Nanjing 210093, China

[2] Laboratory for Shock Wave and Detonation Physics, Institute of Fluid Physics, China Academy of Engineering Physics, Mianyang 621900, China

[3] School of Physical Science and Technology, Shanghai Tech University, Shanghai 201210, China

[4] Beijing National Laboratory for Condensed Matter Physics, Institute of Physics, Chinese Academy of Sciences, Beijing 100190, China

These authors contributed equally: Yang-Yang Lv, Jinlong Xu

Corresponding authors:

longno.2@163.com; shyao@nju.edu.cn; xiezhenda@nju.edu.cn; ybchen@nju.edu.cn

In the supplementary information, we summarized the details of following issues:

Supplementary Note 1: Electrical/magneto-transport properties of β-WP_2 crystals.

Supplementary Note 2: Comparison of HHG in β-WP_2 with representative HHG on other solid materials.

Supplementary Note 3: Dependence of HHG on pump intensity for β-WP_2 crystals.

Supplementary Note 4: The calculated electronic band structure and Berry curvature of β-WP_2.

Supplementary Note 5: Refractive index of β-WP_2 crystals.

Supplementary Note 6: Other possible mechanisms leading to HHG.

Supplementary Note 7: Fitting the harmonic intensity versus polarizer rotation angle for 2nd HHG.

Supplementary Note 8: Simulations of polarization-dependent odd- and even-order HHG intensity.

Supplementary Note 1: Electrical/magneto-transport properties of β-WP_2 crystals

As shown in Supplementary Fig. 1a, ρ_{xx} of β-WP_2 single crystals exhibits a metallic behavior. Quantitatively, ρ_{xx} are 3.27×10^{-4} and 6.68×10^{-8} Ω·mm at 300 and 2 K, respectively, showing an extremely high conductivity. The temperature-dependent carrier concentrations and mobilities are calculated by fitting the magnetoresistance (MR) and Hall data with the two-carrier model (as displayed in Supplementary Fig. 1d). Quantitatively, $n_e = 4.16\times10^{21}$ cm^{-3} and $n_h = 4.17\times10^{21}$ cm^{-3}, $\mu_e = 2.49\times10^5$ cm^2 V^{-1} s^{-1} and $\mu_h = 2.68\times10^5$ cm^2 V^{-1} s^{-1} at 2 K, respectively.

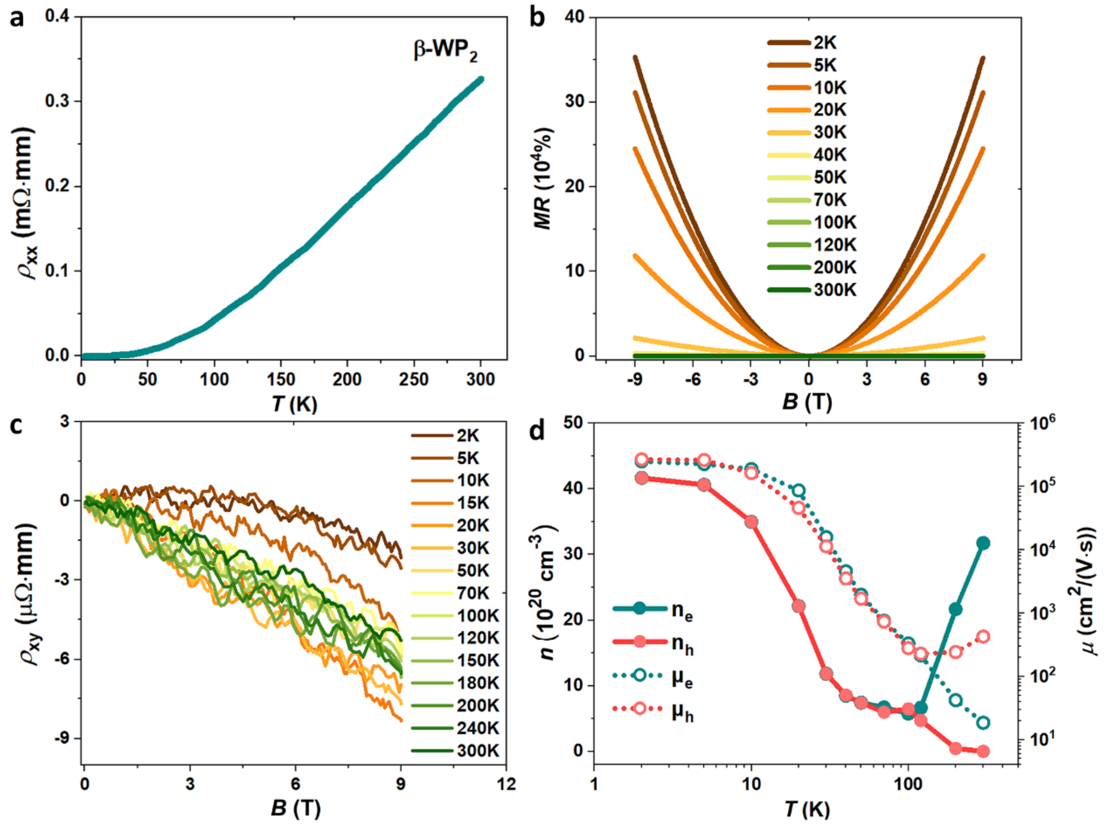

Supplementary Fig. 1. a, Temperature-dependent resistivity of ρ_{xx} for β-WP_2 single crystals in a temperature range of 2-300 K with the electric current *I* along the *a* axis. **b,** The magnetic field dependence of magnetoresistance (MR) for β-WP_2 single crystals at various temperatures with *I//a* axis and *B//b* axis. **c,** Hall resistivity ρ_{xy} as functions of *B//b* axis for β-WP_2 single crystal with temperatures ranging from 2 to 300 K. **d,** The dependence of charge carrier densities n_e and n_h, as well as carrier mobility μ_e and μ_h of electrons and holes on temperature, respectively. These values were extracted from fitting the MR and Hall data by the two-carrier model.

Supplementary Note 2: Comparison of HHG in β-WP_2 with representative HHG on other solid materials

Supplementary Table 1. Comparison of HHG in β-WP_2 crystals with representative HHG on other solid materials.

Materials	Pump wavelength (μm)	Maximal harmonic Order (λ, nm)	Harmonics modes	I_{min} for odd order (TW/cm^2)	I_{min} for even order (TW/cm^2)	Reference
**Solid Ar**	1.333	35th (38.2)	Odd	~3 (13th)	—	[1]
**Solid Kr**	1.333	37th (36)	Odd	~11 (13th)	—	[1]
**Graphene**	4.77	9th (530)	Odd	~0.09 (5th)<br>~0.8 (9th)	—	[2]
**MgO**	1.3	21th (62)	Odd	<1.7 (3rd)	—	[3]
	1.32	19th (69)	Odd	<20 (9th)	—	[4]
**Nanostructured ZnO**	2.0	9th (222)	Odd	0.03(5th)	—	[5]
**Silicon**	2.1	9th (233)	Odd	0.06 (9th)	—	[6]
**SiO_2**	~0.6-0.8	17th (40)	Odd	~13 (11th)	—	[7]
**Fused SiO_2**	~1.3	32th (50)	Odd	1.4	—	[8]
**Quartz**	~0.7	~13th (54)	Odd	10 (13th)	—	[9]
**Sapphire**	0.8	13th (61)	Odd	0.366 (7th)	—	[10]
	0.8	13th (62)	Odd	3.2 (7th)	—	[11]
**Perovskite**	3.5	13th (270)	Odd	<0.5 (11th)	—	[12]
**C_{60}**	0.8	25th (32)	Odd	~100 (25th)	—	[13]
**metal-sapphire nanostructure**	0.8	13th (60)	Odd (and uncertain 8th)	~0.1 (13th)	—	[14]
**Cd_3As_2**	0.3 THz	7th (2.1 THz)	Odd	~6×10^{-6} (7th)	—	[15]
**ZnO**	3.5	13th (270)	Odd	~1 (13th)	—	[16]
	3.25	25th (130)	Odd and even	<0.6 (11th)	<9 (12th)	[17]
**MoS_2**	4.13	13th (318)	Odd and even	<0.9 (9th)	<1.3(12th)	[18]
**a-quartz**	0.8	22th (36)	Odd and even	—	>10 (22th)	[19]
**Si-sapphire**	2.2	11th (220)	Odd and even	~1.1 (7th)	~2.8 (10th)	[20]
**GaSe**	30 THz	22th (675THz)	Odd and even	~13 (21th)	~13 (22th)	[21]
	5	11th (460)	Odd and even	~0.27 (11th)	~0.27 (10th)	[22]
**β-WP_2**	1.9	10th (190)	Odd and even	0.23 (9th)	0.29 (10th)	This work

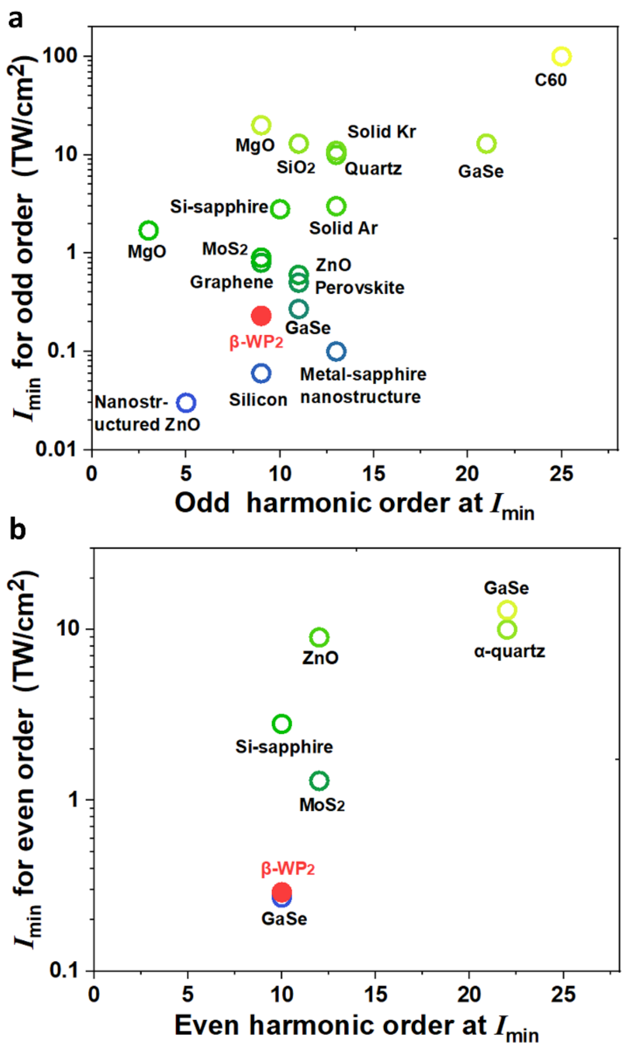

Supplementary Fig. 2. The threshold of pump intensity for odd- (**a**) and even-order (**b**) HHG of different solid materials.

Supplementary Note 3: Dependence of HHG on pump intensity for β-WP_2 crystals

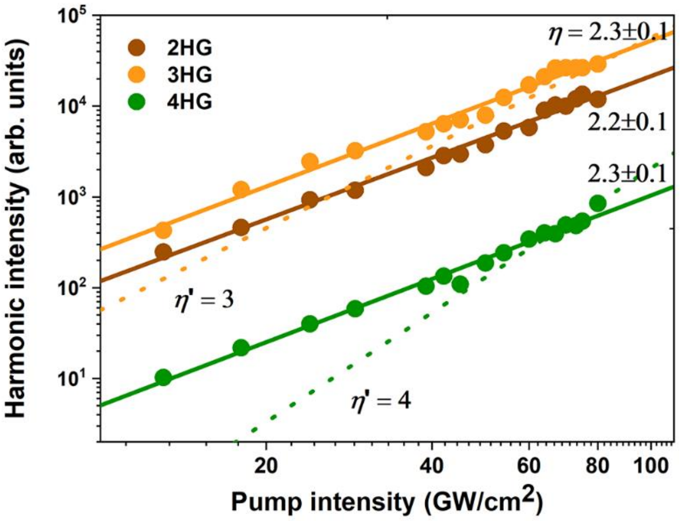

Supplementary Fig. 3. Dependence of HHG on pump intensity for β-WP_2 single crystals. The measured harmonic yield as a function of the peak pump intensity I for harmonic orders h=2, 3, and 4 (dots).

Supplementary Note 4: The calculated electronic band structure and Berry curvature of β-WP_2

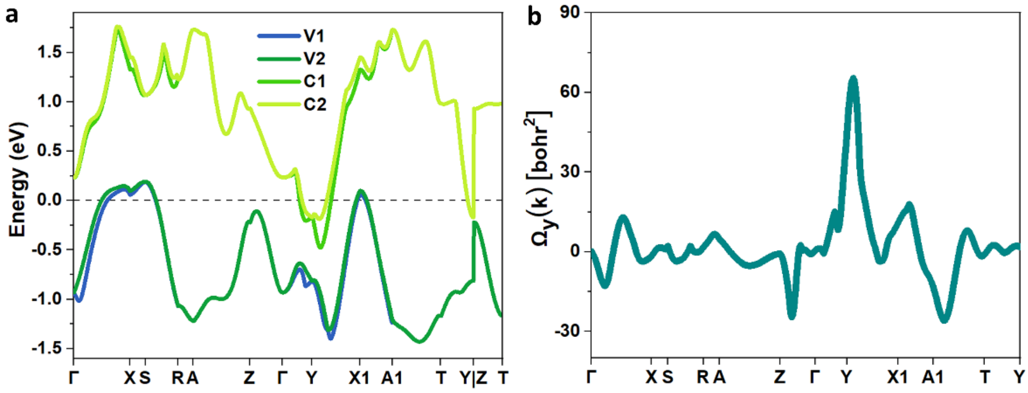

Supplementary Fig. 4. a, Calculated electronic band structure of β-WP_2 near the Fermi Energy. **b,** Theoretically calculated magnitude of the Berry curvature $|\Omega|$ of β-WP_2.

Supplementary Note 5: Refractive index of β-WP_2 crystals

The normal refractive index and extinction coefficient of β-WP_2 were measured by ellipsometry and shown in Supplementary Fig. 5. By means of extinction coefficient of β-WP_2 at 1900 nm, the penetration depth of β-WP_2 can be calculated as 252 nm. It suggests that HHG observed in our work comes from bulk effect of β-WP_2 crystals, rather than surface effect.

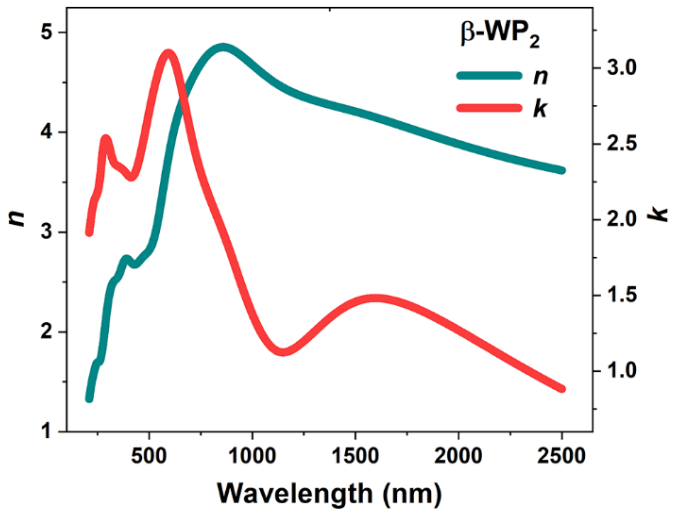

Supplementary Fig. 5. The dielectric constant and refractive index of β-WP_2 crystals.

Supplementary Note 6: Other possible mechanisms leading to HHG

6.1 Rule out even-order HHG coming from perturbative nonlinear optics

In the viewpoint of nonlinear optics, if the crystals have non-centrosymmetric structure, the nonlinear electrical polarization P can be written as:

$$P = \chi^{(1)}E + \chi^{(2)}E^2 + \chi^{(3)}E^3 + \cdots$$

We do can observe the high-order harmonic generation (HHG), especially even-order $\chi^{(2N)}$ is not zero if there is no inversion symmetry. And we can see that intensity

of second-harmonic generation is still proportional to the square of incident intensity. But if the order is higher than two, the intensity of corresponding n-order harmonic generation will proportional to I_0^{n} . For example, the intensity of fourth-harmonic generation $I_4 \propto I_0{}^4$. In our experiment, we found that the intensity of n-order-harmonic generation in β-WP_2 crystals is approximatively proportional to $I_0{}^x$, power index x ranged from 2.1 to 2.5. Obviously, it is different from 2N-power in perturbative nonlinear optics. Therefore, we rule out the possibility of the conventional nonlinear optical process in our even-order HHG experiment.

6.2 Rule out the HHG coming from interband-transition

In some papers, it has been proposed that the inter-band transition and successive the Bloch oscillation in conduction band leads to the generation of both even- and odd-order HHG. But there are two reasons to rule out the inter-band transition mechanism:

6.2.1 Ultrafast transient absorption study of interband dynamics in β-WP_2

Ultrafast transient absorption (TA) spectroscopy is a precise technique to investigate the interband dynamic process of materials. Here, we directly measured the dynamic process of interband TA probed at 1900 nm (~100 fs pulse duration, 1 kHz repetition rate) with pumped at 800 nm (800-nm photons can excite all transition whose energy is lower than 1.55 eV) in a homemade femtosecond pump-probe system to identify the probability of interband transition in β-WP_2 system. As shown in Supplementary Fig. 6, along S direction, close Z point along the T-Z direction and along gamma-Y direction, there are direct bandgaps of 0.6~0.8 eV between the valence band edge and the conduction band bottom. And the photon energy of 800-nm pump is sufficient to across the band gap. If there is obvious interband transition at the points along S direction, along the T-Z direction and along gamma-Y direction under 1900 nm photons, clear decay in TA response on 1900-nm probe pulse should be detected.

However, as shown in Supplementary Fig. 7, we did not detect any discernible decay response at 1900 nm, even under the pump strength of 6.2 GW/cm^2. For comparison, we also carried out the TA at several shorter probe wavelengths under the same 800-nm pump intensity, as shown in Supplementary Fig. 7. One can see the distinct absorption response at 1000 nm, which means that there is stronger absorption than 1900 nm case. These results confirm the weak interband transition at 1900 nm.

To cross-check above experimental data, we calculated the imaging part $\varepsilon_1(E)$ of dielectric constant $\varepsilon(E)$ (E is the energy, and can be converted to wavelength of electromagnetic-wave by timing 1240 nm/eV) by first-principles LDA method. The data is presented in Supplementary Fig. 8. One can see that the ε_1 at 0.65 eV is as small as 5.2, in contrast, there are a giant Drude peak at zero-energy and ε_1 being as large as 20.0 at 2.1 eV. We also can see that theoretical ε_1 is in line with TA experiment shown in Supplementary Fig. 7, absorption at 1000 nm is larger than that at 1900 nm.

Combining above two data, we do believe that the inter-band transition of β-WP_2 is quite weak and its role in observed HHG is *immaterial*. Therefore, the efficient HHG in this work mainly comes from the intraband Bloch oscillation of free electron on overlapped conduction and valence bands of β-WP_2 (semimetals).

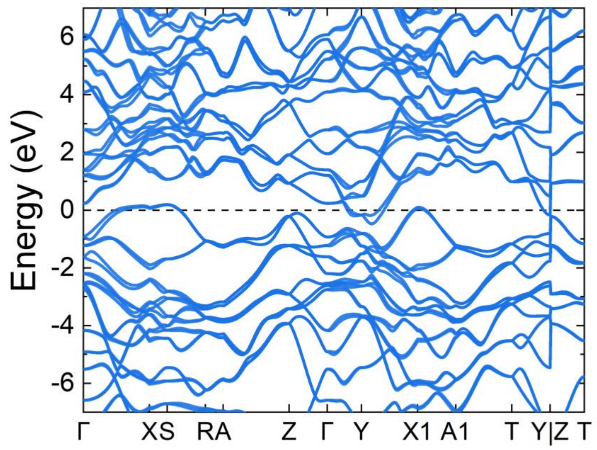

Supplementary Fig. 6. Electronic band structure of β-WP_2 considering spin-orbit coupling with the energy range of -7.0~7.0 eV.

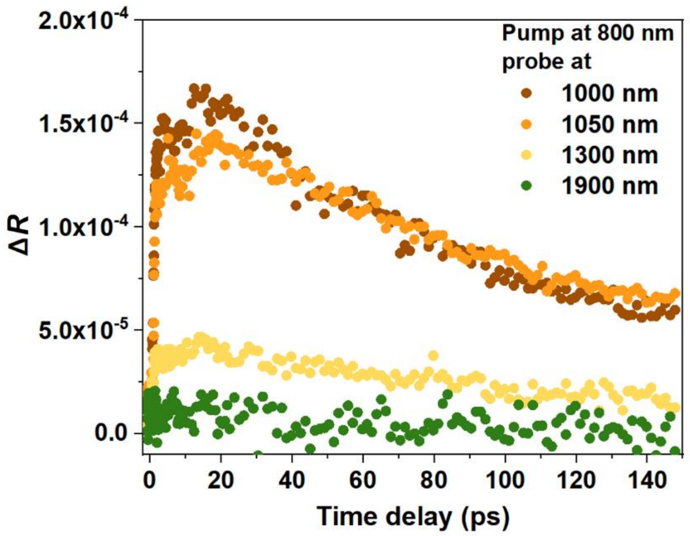

Supplementary Fig. 7. Comparison of transient absorption of β-WP_2 at different wavelengths. One can see that the small Δabsorption at 1900 nm. The 800-nm pump intensity is 6.2 GW/cm^2, and the probe intensities at different wavelengths are fixed at 0.6 GW/cm^2.

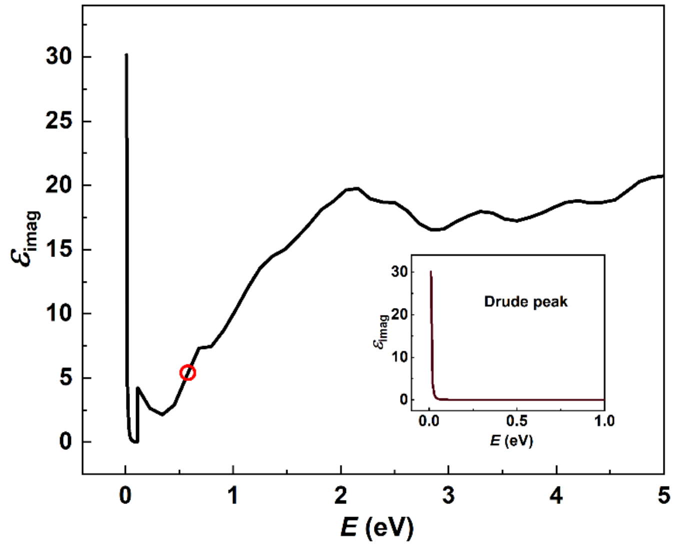

Supplementary Fig. 8. Theoretical imaginary part of dielectric constant ε_{imag}-energy (E) relationship. One can see that at this energy range, dielectric absorptions have two peaks, one is the Drude peak at 0-energy (ε_{imag}=30); the other is the peak at 2.1 eV (ε_{imag}=20.0). ε_{imag} at 0.65 eV (corresponding electromagnetic-wavelength of 1900 nm used in our HHG experiment) is around 5.2.

6.2.2 Mechanism of HHG in metals is different from semiconductors

Generally, in semiconductor, both the interband polarization and the intraband current could have contribution to HHG. But, the metals may have different situation compared to semiconductor. Recently, Shimomura, K. et al., have reported the HHG in metal is mainly attributed to intraband radiation because there is a high-density of free electron in metals, quite similar to our β-WP_2 case (~ 10^{21} cm^{-3}) [23]. In other words, the interband polarization would plays minor role in HHG of metals. As shown in Supplementary Fig. 6, the electronic band structure of β-WP_2 is sure a typical semimetal within a large energy range (-7.0~7.0 eV).

Considering all these two items, we think that the inter-band transition contributes little to both odd- and even-order HHG in β-WP_2.

6.3 Even-order HHG coming from Berry curvature mechanism

In some papers, the even harmonics generation is attributed to nonvanishing Berry curvature in the electronic bands of the materials with the non-centrosymmetric structure. In Table S1, we found 6 papers that reported the observation of even-harmonic-generation in semiconductors, 3 papers have discussed the even-harmonic generation coming from the Berry curvature [17-19]. In our work, we took the Berry curvature mechanism for the even harmonics. There are two reasons: 1) It has been established that there are Weyls points in β-WP_2 crystals and corresponding Berry curvature by angle-resolved photoemission spectroscopy and theoretical calculations [24,25]. In this condition, the kinetic equations of Bloch electrons naturally have the term of Berry curvature. 2) We can see that the intensity of even-order (for example second-order) is quite comparable to odd-order (third harmonic generation) in our experiment. This feature is also used to support the even-order HHG coming from Berry curvature mechanism in some papers [18,19].

Notably, based on the above discussions, we think that the mechanism of even-

order harmonic generation attributed to Berry curvature is the most natural explanation for our experimental observation.

Supplementary Note 7: Fitting the harmonic intensity versus polarizer rotation angle for 2nd HHG

As shown in Supplementary Fig. 9, we can see that 2nd HHG, different from other high-order (≥3) HHG, comes from both perturbative nonlinear second-harmonic generation and Berry curvature effect.

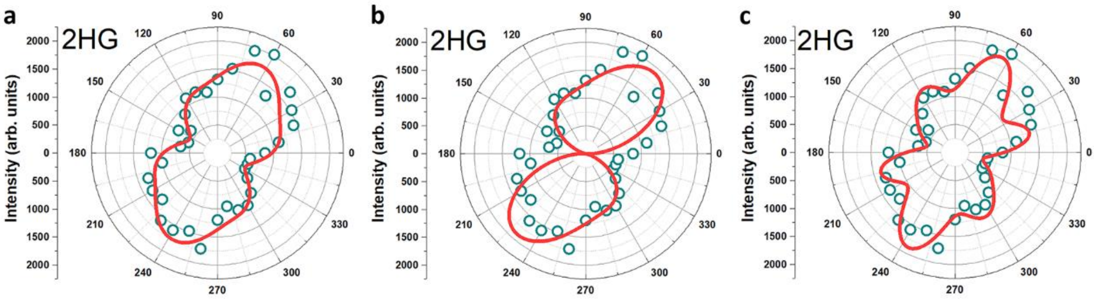

Supplementary Fig. 9. The harmonic intensity versus polarizer rotation angle for harmonic order h=2. The red lines are the fitting curves based on: **a,** symmetry of the *ac*-plane (point group, mm2); **b,** Berry curvature; **c,** both symmetry of the *ac*-plane and Berry curvature.

Supplementary Note 8: Simulations of polarization-dependent odd- and even-order HHG intensity

Consider linear polarization

$$\mathbf{A}(\theta,t)=\left[A_0\cos\theta\mathbf{e_z}+A_0\sin\theta\mathbf{e_x}\right]G(t)\sin(\omega_L t), \quad \text{(S1)}$$

where $A_0,\theta,\omega_L,G(t)$ are the peak, angular with (010) axis, angular frequency, the envelope of the laser vector potential. And $\mathbf{e_x},\mathbf{e_z}$ are the unit vectors along *x*- and *z*-direction.

In the simple case of a simple sine field $\left[G(t)=1\right]$, the polarization-dependent field can be written as:

$$\mathbf{E}(\theta,t)=-\frac{\partial\mathbf{A}(\theta,t)}{\partial t}=-A_0\omega_{\mathrm{L}}\left[\cos\theta\mathbf{e}_{\mathbf{z}}+\sin\theta\mathbf{e}_{\mathbf{x}}\right]\cos(\omega_{\mathrm{L}}t)\,. \tag{S2}$$

$$\hbar\frac{d\mathbf{k}(t)}{dt}=-e\mathbf{E}(t)=e\frac{\partial\mathbf{A}(t)}{\partial t} \tag{S3}$$

$$\mathbf{k}(t)=\mathbf{k}(0)+\frac{e}{\hbar}\mathbf{A}(t) \tag{S4}$$

S3 and S4 are the time-evolution crystal momentum under stimulus of electromagnetic field. According to Ref. [19], in a typical semi-classical model, it can be assumed k(0) as zero, thus crystal momenta k_x and k_y at time t can be written as :

$$\begin{aligned}k_x(t)&=\frac{eA_0}{\hbar}\sin\theta\sin(\omega_{\mathrm{L}}t)\\ k_z(t)&=\frac{eA_0}{\hbar}\cos\theta\sin(\omega_{\mathrm{L}}t)\end{aligned}\,. \tag{S5}$$

Equation (S5) can be used to calculate the intensity of odd-order HHG of β-WP_2 described in the main text.

In what follows, we described the formula to calculate the group velocity of even-order HHG of β-WP_2. According to the definition of the group velocity with anomalous term contributed by Berry curvature, group velocity can be written as:

$$\mathbf{v}(\mathbf{k})=\underbrace{\frac{1}{\hbar}\frac{\partial\varepsilon(\mathbf{k})}{\partial\mathbf{k}}}_{\mathbf{v}_{\mathrm{odd}}}-\underbrace{\dot{\mathbf{k}}\times\Omega(\mathbf{k})}_{\mathbf{v}_{\mathrm{even}}}\,. \tag{S6}$$

For the first term, consider energy band with the tight-bonding approximation in the *ac*-plane

$$\varepsilon(\mathbf{k})=\varepsilon_0-2[t_{x,1}\cos(k_xa)+t_{z,1}\cos(k_za)+t_{x,2}\cos(2k_xa)+t_{z,2}\cos(2k_za)] \tag{S7}$$

where $\varepsilon_0,a,k_\alpha,t_{\alpha,i}$ is the band offset, lattice constant, the crystal momentum, and the hopping integral values with $\alpha=x,z;\quad i=1,2$.

According to the equations (S5), (S6) and (S7), the first term of time-dependent group velocity of the Bloch electronic in equation (S6) can be written as:

$$\mathbf{v}_{\text{odd}}(t)=\frac{2a}{\hbar}\left[t_{x,1}\sin(k_x a)+2t_{x,2}\sin(2k_x a)\right]\mathbf{e}_x+\frac{2a}{\hbar}\left[t_{z,1}\sin(k_z a)+2t_{z,2}\sin(2k_z a)\right]\mathbf{e}_z$$
$$=\frac{2a}{\hbar}\left[t_{x,1}\sin(\frac{eA_0a}{\hbar}\sin\theta\sin(\omega_{\text{L}}t))+2t_{x,2}\sin(\frac{2eA_0a}{\hbar}\sin\theta\sin(\omega_{\text{L}}t))\right]\mathbf{e}_x \qquad .(\text{S8})$$
$$+\frac{2a}{\hbar}\left[t_{z,1}\sin(\frac{eA_0a}{\hbar}\cos\theta\sin(\omega_{\text{L}}t))+2t_{z,2}\sin(\frac{2eA_0a}{\hbar}\cos\theta\sin(\omega_{\text{L}}t))\right]\mathbf{e}_z$$

Apply the Jacobi-Anger expansion, and equation (S8) can be written as:

$$\mathbf{v}_{\text{odd}}(t)=\frac{4a}{\hbar}\left\{t_{x,1}\sum_{M=1}^{\infty}\left[J_{2M-1}\left(\frac{eA_0a}{\hbar}\sin\theta\right)+\frac{2t_{x,2}}{t_{x,1}}J_{2M-1}\left(\frac{2eA_0a}{\hbar}\sin\theta\right)\right]\sin((2M-1)\omega_{\text{L}}t)\right\}\mathbf{e}_x \qquad .(\text{S9})$$
$$+\frac{4a}{\hbar}\left\{t_{z,1}\sum_{M=1}^{\infty}\left[J_{2M-1}\left(\frac{eA_0a}{\hbar}\cos\theta\right)+\frac{2t_{z,2}}{t_{z,1}}J_{2M-1}\left(\frac{2eA_0a}{\hbar}\cos\theta\right)\right]\sin((2M-1)\omega_{\text{L}}t)\right\}\mathbf{e}_z$$

Similarly we can expand the second term of equation (S6) by using the Fourier expansion of $\mathbf{\Omega}(\mathbf{k})$:

$$\mathbf{\Omega}(\mathbf{k})=\left[\gamma_{x,1}sin(k_x a)+\gamma_{x,2}sin(2k_x a)+\gamma_{z,1}sin(k_z a)+\gamma_{z,2}sin(2k_z a)\right]\mathbf{e}_y. \qquad (\text{S10})$$

And then it will arrive at

$$v_{\text{even}}(t)=\dot{\mathbf{k}}(t)\times\mathbf{\Omega}(\mathbf{k})$$
$$=\frac{eA_0\omega_{\text{L}}}{\hbar}\cos(\omega_{\text{L}}t)\left[\cos\theta\mathbf{e_z}+\sin\theta\mathbf{e_x}\right]\times\Omega_y(\mathbf{k})\mathbf{e_y} \qquad (\text{S11})$$
$$=\frac{eA_0\omega_{\text{L}}}{\hbar}\underbrace{\Omega_y(\mathbf{k})\cos(\omega_{\text{L}}t)}_{\mathbf{T}(t)}\left[-\cos\theta\mathbf{e_x}+\sin\theta\mathbf{e_z}\right]$$

where the time-dependent term can be:

$$\mathbf{T}(t)=\cos(\omega_{\text{L}}t)\left[\sum_{n=1}^{2}\gamma_{x,n}\sin\left(\frac{neA_0a}{\hbar}\sin\theta\sin(\omega_{\text{L}}t)\right)\right.$$
$$\left.+\sum_{n=1}^{2}\gamma_{z,n}\sin\left(\frac{neA_0a}{\hbar}\cos\theta\sin(\omega_{\text{L}}t)\right)\right]$$
$$=2\sum_{n=1}^{2}\sum_{M=1}^{\infty}\left[\gamma_{x,n}J_{2M-1}\left(\frac{neA_0a}{\hbar}\sin\theta\right)\sin\left[(2M-1)\omega_{\text{L}}t\right]\cos(\omega_{\text{L}}t)\right.$$
$$\left.+\gamma_{z,n}J_{2M-1}\left(\frac{neA_0a}{\hbar}\cos\theta\right)\sin\left[(2M-1)\omega_{\text{L}}t\right]\cos(\omega_{\text{L}}t)\right] \qquad (\text{S12})$$
$$=\sum_{n=1}^{2}\sum_{M=1}^{\infty}\left\{\gamma_{x,n}J_{2M-1}\left(\frac{neA_0a}{\hbar}\sin\theta\right)\left[\sin(2M\omega_{\text{L}}t)-\sin(2(M-1)\omega_{\text{L}}t)\right]\right.$$
$$\left.+\gamma_{z,n}J_{2M-1}\left(\frac{neA_0a}{\hbar}\cos\theta\right)\left[\sin(2M\omega_{\text{L}}t)-\sin(2(M-1)\omega_{\text{L}}t)\right]\right\}$$

The source term of radiation in the wave equation is given by time derivative of

the current density, and is given by

$$I(t) \propto \left| \frac{\partial J(t)}{\partial t} \right| \propto \left| \frac{\partial v(t)}{\partial t} \right|^2 , \tag{S13}$$

which yields the following expression in the frequency domain

$$I(\omega) \propto \left| \omega v(\omega) \right|^2 . \tag{S14}$$

According to the equations (S9), (S11), (S12) and (S14), the odd-order HHG intensity is

$$\begin{aligned} I_{2M-1} \propto \left[(2M-1)\omega_{\mathrm{L}} \frac{4a}{\hbar} t_{x,1} \right]^2 & \left\{ \left[J_{2M-1}\left(\frac{eA_0 a}{\hbar} \sin\theta \right) + \frac{2t_{x,2}}{t_{x,1}} J_{2M-1}\left(\frac{eA_0 a}{\hbar} 2\sin\theta \right) \right]^2 \right. \\ & \left. + (\frac{t_{z,1}}{t_{x,1}})^2 \left[J_{2M-1}\left(\frac{eA_0 a}{\hbar} \cos\theta \right) + \frac{2t_{z,2}}{t_{z,1}} J_{2M-1}\left(\frac{eA_0 a}{\hbar} 2\cos\theta \right) \right]^2 \right\} \end{aligned} . \tag{S15}$$

the even-order HHG intensity is

$$\begin{aligned} I_{2M} \propto (2M\omega_{\mathrm{L}}{}^2 \frac{e\mathrm{A}_0}{\hbar} \gamma_{x,1})^2 & \left\{ \left[J_{2M-1}\left(\frac{eA_0 a}{\hbar} \sin\theta \right) - J_{2M+1}\left(\frac{eA_0 a}{\hbar} \sin\theta \right) \right] \right. \\ & + \frac{\gamma_{x,2}}{\gamma_{x,1}} \left[J_{2M-1}\left(\frac{eA_0 a}{\hbar} 2\sin\theta \right) - J_{2M+1}\left(\frac{eA_0 a}{\hbar} 2\sin\theta \right) \right] \\ & + \frac{\gamma_{z,1}}{\gamma_{x,1}} \left[\left(J_{2M-1}\left(\frac{eA_0 a}{\hbar} \cos\theta \right) - J_{2M+1}\left(\frac{eA_0 a}{\hbar} \cos\theta \right) \right) \right. \\ & \left. \left. + \frac{\gamma_{z,2}}{\gamma_{z,1}} \left(J_{2M-1}\left(\frac{eA_0 a}{\hbar} 2\cos\theta \right) - J_{2M+1}\left(\frac{eA_0 a}{\hbar} 2\cos\theta \right) \right) \right] \right\}^2 \end{aligned} . \tag{S16}$$

The equations (S15) and (S16) are equations to calculate the intensity of HHG of β-WP_2, and retrieve the hopping integrals and expansion coefficients of Berry curvature of electronic band structure of β-WP_2.

Supplementary References

[1] Ndabashimiye, G. et al. Solid-state harmonics beyond the atomic limit. *Nature* **534**, 520-523 (2016).

[2] Yoshikawa, N., Tamaya, T. & Tanaka, K. High-harmonic generation in graphene enhanced by elliptically polarized light excitation. *Science* **356**, 736-738 (2017).

[3] You, Y. S., Reis, D. A. & Ghimire, S. Anisotropic high-harmonic generation in bulk crystals. *Nat. Phys.* **13**, 345-349 (2017).

[4] J Lu, J., Cunningham, E. F., You, Y. S., Reis, D. A. & Ghimire, S. Interferometry of dipole phase in high harmonics from solids. *Nat. Photon.* **13**, 96-100 (2019).

[5] Sivis, M. et al. Tailored semiconductors for high-harmonic optoelectronics. *Science* **357**, 303-306 (2017).

[6] Klemke, N. et al. Polarization-state-resolved high-harmonic spectroscopy of solids. *Nat. Commun.* **10**, 1319 (2019).

[7] Luu, T. T. et al. Extreme ultraviolet high-harmonic spectroscopy of solids. *Nature* **521**, 498-502 (2015).

[8] You, Y. S. et al. High-harmonic generation in amorphous solids. *Nat. Commun.* **8**, 724 (2017).

[9] Garg, M., Kim, H. Y., & Goulielmakis, E. Ultimate waveform reproducibility of extreme-ultraviolet pulses by high-harmonic generation in quartz. *Nat. Photon.* **12**, 291-296 (2018).

[10] Kim, H., Han, S., Kim, Y. W., Kim, S. & Kim, S. -W. Generation of Coherent Extreme-Ultraviolet Radiation from Bulk Sapphire Crystal. *ACS Photon.* **4**, 1627-1632 (2017).

[11] Han, S. et al. Extraction of higher-order nonlinear electronic response in solids using high harmonic generation. *Nat. Commun.* **10**, 3272 (2019).

[12] Hirori, H. et al. High-order harmonic generation from hybrid organic-inorganic perovskite thin films. *APL Mater.* **7**, 041107 (2019).

[13] Ganeev, R. A. et al. Higher-Order Harmonic Generation from Fullerene by Means of the Plasma Harmonic Method. *Phys. Rev. Lett.* **102**, 013903 (2009).

[14] Han, S. et al. High-harmonic generation by field enhanced femtosecond pulses in

metal-sapphire nanostructure. *Nat. Commun.* **7**, 13105 (2016).

[15] Kovalev, S. et al. Non-perturbative terahertz high-harmonic generation in the three-dimensional Dirac semimetal Cd_3As_2. *Nat. Commun.* **11**, 2451 (2020).

[16] Wang, Z. et al. The roles of photo-carrier doping and driving wavelength in high harmonic generation from a semiconductor. *Nat. Commun.* **8**, 1686 (2017).

[17] Ghimire, S. et al. Observation of high-order harmonic generation in a bulk crystal. *Nat. Phys.* **7**, 138-141 (2011).

[18] Liu, H. Z. et al. High-harmonic generation from an atomically thin semiconductor. *Nat. Phys.* **13**, 262-265 (2017).

[19] Luu, T. T. & Wörner, H. J. Measurement of the Berry curvature of solids using high-harmonic spectroscopy. *Nat. Commun.* **9**, 916 (2018).

[20] Vampa, G., Liu, H. Z., Heinz, T. F. & Reis, D. A. Disentangling interface and bulk contributions to high-harmonic emission from solids. *Optica* **6**, 553-556 (2019).

[21] Schubert, O. et al. Sub-cycle control of terahertz high-harmonic generation by dynamical Bloch oscillations. *Nat. Photon.* **8**, 119-123 (2014).

[22] Kaneshima, K. et al. Polarization-Resolved Study of High Harmonics from Bulk Semiconductors. *Phys. Rev. Lett.* **120**, 243903 (2018).

[23] Shimomura, K. et al. High Harmonic Generation in Metallic Phase of 2H-$NbSe_2$. 2019 44th International Conference on Infrared, Millimeter, and Terahertz Waves (IRMMW-THz), Paris, France, 2019, pp. 1-2, doi: 10.1109/IRMMW-THz.2019.8874306.

[24] Kumar, N. et al. Extremely high magnetoresistance and conductivity in the type-II Weyl semimetals WP_2 and MoP_2. *Nat. Commun.* **8**, 1642 (2017).

[25] Zhang, K. X. et al. Butterfly-Like Anisotropic Magnetoresistance and Angle-Dependent Berry Phase in a Type-II Weyl Semimetal WP_2. *Chin. Phys. Lett.* **37**(9), 090301 (2020).
